# Stevens-Johnson Syndrome Associated with Drugs and Vaccines in Children: A Case-Control Study

**DOI:** 10.1371/journal.pone.0068231

**Published:** 2013-07-16

**Authors:** Umberto Raucci, Rossella Rossi, Roberto Da Cas, Concita Rafaniello, Nadia Mores, Giulia Bersani, Antonino Reale, Nicola Pirozzi, Francesca Menniti-Ippolito, Giuseppe Traversa, Italian Multicenter Study Group for Vaccine Safety in Drug and Children

**Affiliations:** 1 Paediatric Emergency Department, Bambino Gesù Children’s Hospital, IRCCS, Roma, Italy; 2 National Centre for Epidemiology, Surveillance and Health Promotion, National Institute of Health, Roma, Italy; 3 Department of Experimental Medicine, Section of Pharmacology “L. Donatelli”, Second University of Napoli, Napoli, Italy; 4 Pharmacology and Paediatrics, Università Cattolica S. Cuore, Roma, Italy; Nottingham University, United Kingdom

## Abstract

**Objective:**

Stevens-Johnson Syndrome (SJS) is one of the most severe muco-cutaneous diseases and its occurrence is often attributed to drug use. The aim of the present study is to quantify the risk of SJS in association with drug and vaccine use in children.

**Methods:**

A multicenter surveillance of children hospitalized through the emergency departments for acute conditions of interest is currently ongoing in Italy. Cases with a diagnosis of SJS were retrieved from all admissions. Parents were interviewed on child’s use of drugs and vaccines preceding the onset of symptoms that led to the hospitalization. We compared the use of drugs and vaccines in cases with the corresponding use in a control group of children hospitalized for acute neurological conditions.

**Results:**

Twenty-nine children with a diagnosis of SJS and 1,362 with neurological disorders were hospitalized between 1^st^ November 1999 and 31^st^ October 2012. Cases were more frequently exposed to drugs (79% vs 58% in the control group; adjusted OR 2.4; 95% CI 1.0–6.1). Anticonvulsants presented the highest adjusted OR: 26.8 (95% CI 8.4–86.0). Significantly elevated risks were also estimated for antibiotics use (adjusted OR 3.3; 95% CI 1.5–7.2), corticosteroids (adjusted OR 4.2; 95% CI 1.8–9.9) and paracetamol (adjusted OR 3.2; 95% CI 1.5–6.9). No increased risk was estimated for vaccines (adjusted OR: 0.9; 95% CI 0.3–2.8).

**Discussion:**

Our study provides additional evidence on the etiologic role of drugs and vaccines in the occurrence of SJS in children.

## Introduction

Adverse drug reactions (ADRs) in children are an important public health problem. Two systematic reviews of observational studies on ADRs causing paediatric hospitalizations showed an estimated admission rate of 2.1% (95% CI 1.0%–3.8%) between 1976 and 1996 [1], and of 2.9% (95% CI 2.6%–3.1%) in the period from 1964 to 2009 [2].

Cutaneous ADRs are the most frequently reported ADRs both in adults and children [3]. These events are generally mild and self-limiting, although in rare instances they can be life threatening [4], [5]. Stevens-Johnson Syndrome (SJS) and Toxic Epidermal Necrolysis (TEN) are the most serious conditions, characterized by extensive detachment of epidermis and erosions of mucous membranes [4], [6]. The two conditions are considered to differ in their severity, with the extent of skin detachment being <10% for SJS, >30% for TEN and between 10 and 30% for so called SJS-TEN overlap. The estimated incidence is 1–7 cases per million person-years for SJS and 0.4–1.5 cases per million person-years for TEN [7].

In adults, between 60% and 70% of SJS and TEN was attributed to drug use [8], [9]. However, the overall evidence concerning the relationship between drugs and the occurrence of SJS and TEN in children is still limited, because of the rarity of the disease and the scarcity of observational studies. The most relevant findings derive from a pooled analysis [10] that focused on children (age <15 years) enrolled in two large case-control studies conducted in the general population [11], [12]. Drugs strongly associated with the occurrence of SJS and TEN were anti-infective sulphonamides, phenobarbital, carbamazepine and lamotrigine. An increased risk was also observed for valproic acid, non-steroidal anti-inflammatory drugs (NSAIDs), and paracetamol [10].

Since 1999 an active surveillance aimed at ascertaining the role of drugs and vaccines in the occurrence of specific acute conditions responsible for the hospitalization of paediatric patients has been conducted in Italy [13], [14], [15]. Non-infective muco-cutaneous diseases were among the conditions included in the surveillance and we focused on cases of SJS and TEN to contribute further information on the causal role of drugs and vaccines. The aim of the study was to provide risk estimates of medicines that are already known to be associated with SJS or TEN and to point out potential new signals.

## Methods

### Ethics Statement

The protocol of the study was approved by the Ethical Committee of the participating clinical centres: Regina Margherita Children’s Hospital (Torino), Giannina Gaslini Paediatric Hospital (Genova); Department of Paediatrics, University Hospital (Padova); Anna Meyer Children’s University Hospital (Firenze); Department of Paediatrics, University Hospital (Perugia); Pharmacology and Paediatrics and Developmental Neuroscience, Università Cattolica Sacro Cuore (Roma); Emergency Department, Bambino Gesù Children Hospital (Roma); Santobono-Pausilipon Paediatric Hospital (Napoli); Giovanni Di Cristina Paediatric Hospital (Palermo). Written informed consent to use data for research purposes was obtained before conducting parents’ interview.

### Setting and Study Population

The Italian multicentre surveillance on drug and vaccine safety in children is an ongoing active observational study coordinated by the Italian National Institute of Health. The surveillance currently includes nine paediatric hospitals/wards spread throughout the country (Genoa, Turin, Padua, Florence, Perugia, Rome (two hospitals), Naples and Palermo).

The population included in the surveillance consisted of all children (age ≥1 month to 18 years) who visited the emergency department of the participating centers and were hospitalized for one of the following acute conditions: non-infectious muco-cutaneous diseases; neurological disorders; gastroduodenal lesions; thrombocytopenia. General exclusion criteria were represented by a concomitant diagnosis of cancer or immunodeficiency. Within the neurological disorders, febrile seizure, non-incident convulsions (children with a previous diagnosis of epilepsy), and headaches were also excluded.

### Data Collection

Parents were interviewed by a trained pharmacist/physician using a structured questionnaire and data on medical history and exposure to drugs and vaccines were collected. Specifically, drug use was considered of interest for the 3 weeks preceding the onset of symptoms (the index date), whereas the time window for vaccine administration was extended to 6 weeks (12 weeks since October 2009). For all children, the inclusion in the surveillance was based on the clinical diagnosis and was consequently independent on the information on previous exposure to drugs and vaccines. Further details on the study methodology can be found elsewhere [15].

### Definition of Cases and Controls

Cases included children hospitalized through the emergency department for a muco-cutaneous condition who were discharged with a final diagnosis of SJS or TEN. Diagnosis were validated reviewing the clinical records and, among the others, the following information was extracted: site of mucosal lesions (eye, mouth, anus); dermatological consultation; and pharmacological treatments administered during the hospitalization.

Control subjects were all children hospitalized in the participating centers for non-convulsive neurological conditions. These conditions were considered a valid control group, as source population of SJS or TEN cases, since they represent acute conditions admitted, as for the cases, through the emergency departments of the same centres. Children hospitalized for gastroduodenal lesions and for thrombocytopenia were excluded from the control group because these conditions are frequently associated with drug exposure.

### Analysis

Demographic characteristics of SJS patients, together with the exposure to drugs and vaccines preceding the onset of symptoms (index date), are presented on an individual basis. Cases and controls were compared with regard to the exposure to drugs and vaccines before the index date. To take into account the possibility that some drugs may have been prescribed to treat the preliminary symptoms of SJS, a further analysis was conducted after excluding drug use initiated in the index date or in the 2 previous days.

Crude and adjusted Odds Ratios (ORs), with 95% Confidence Intervals (CI), were estimated through a logistic regression model. The potential confounding role of age and concomitant use of any other drug was taken into account in the estimates of the adjusted ORs. Data were analysed with SPSS (v. 17.0).

## Results

Out of 2,483 children hospitalized for a muco-cutaneous condition between 1^st^ November 1999 and 31^st^ October 2012, 37 (1.5%) were hospitalized with a suspected SJS or TEN. We were able to retrieve the clinical records of 35 children (95%). The validation procedure did not confirm 8 cases (22%). The diagnosis of the not confirmed cases were: staphylococcal erythema (3 cases); erythema multiforme (2 cases); Kawasaki disease (1 case); unspecified cutaneous adverse drug reaction (1 case); and cutaneous rash (1 case). Twenty-nine children with a diagnosis of SJS or TEN were analyzed (also including the two children with missing clinical record). In twenty-five cases either a dermatologist or a plastic surgeon was consulted during the hospitalization.

In the same period, 1,362 children diagnosed with neurological disorders were also included in the surveillance. The most frequent diagnosis in the control group were: disturbances of consciousness (such as syncope, faintness, dizziness, somnolence), apparent life threatening events (ALTE), movement disorders (eg. extrapyramidal symptoms) (Table 1). Cases and controls presented no differences in the male to female ratio, whereas cases were younger than controls, with a median age of 3 and 4 years respectively. A positive anamnesis of systemic allergic manifestations was reported in 2 SJS cases (7%) and in 86 children in the control group (6%). The median duration of hospitalization of SJS cases was 9 days (25^th^ to 75^th^ percentile 4–11), three days longer than in the control group.

**Table 1:** Characteristics of the study population.

	Cases	Controls*
Number	29	1,362
Median age (range)	3 y(4 m–12 y)	4 y(1 m–19 y)
% Females	48	50
Anamnestic allergy: N (%)	2 (7)	86 (6)
Median length of hospital stay (days)	9	6
Children exposed to drugs: N (%)	23 (79)	796 (58)
Children exposed to vaccines: N (%)	4 (14)	137 (10)
**Neurological conditions**		
Disturbances of consciousness: N (%)		617 (45)
ALTE: N (%)		160 (12)
Movement disorders: N (%)		133 (10)
Other neurological disorders: N (%)		120 (9)
Peripheral neuropathies: N (%)		110 (8)
Infectious and parainfectiousneuropathies: N (%)		89 (6)
Psychological disorders: N (%)		71 (5)
Vertigo: N (%)		62 (5)

* Neurological conditions.

Only one case of TEN was included, a 48 month old girl exposed to an epicutaneous patch testing for mercury allergy.

Cases were more frequently exposed to drugs in the three weeks preceding the onset of symptoms (79% vs 58% in the control group; OR adjusted by age: 2.4; 95% CI 1.0–6.1). The proportion of children exposed to vaccines were 14% among cases and 10% among controls, even though the difference was almost entirely explained by the difference in the age distribution (adjusted OR 0.9; 95% CI 0.3–2.8).

Only 5 of the 29 cases (17%) did not receive either drugs or vaccines before the index date. The main characteristics of each SJS or TEN case are detailed in Table 2.

**Table 2:** Individual information on SJS and TEN cases.

Sex	Age (months)	Mucosal lesions (Eye, Mouth, Anus)	Treatment (Topical, Supportive, Steroidal)	Drugs or vaccines	Indication	Duration of therapy (days)	Interval between last exposure and index date (days)	Past exposure for acute indications	Exposure during admission
F	4	E, M, A	T, Su	No					
M	5	E, M	T, Su	Salbutamol	Cough	3	0	Yes	Yes
				Fluticasone	Cough	3	0	No	No
				Betamethasone	Cough	3	0	Yes	No
				Amoxicillin+ clavulanic acid	Cough	3	0	Yes	No
M	6	E, M, A	T, Su	Beclomethasone	Dry cough	4	1	No	No
				Hexavalent vaccine	Immunization		23		
F	6	E, M, A	T, Su	No					
M	6	E, A	T, Su	Fluoride	Prophylaxis	10	0	Yes	No
M	12	E, M, A	T, Su	No					
F	13	E, M, A	T, Su	Cephaclor	NR	5	1	Yes	No
				Paracetamol	NR	5	1	Yes	No
				Hexavalent vaccine	Immunization		4		
				Haemophilus influenza vaccine	Immunization		4		
M	13	E, M, A	T, Su, St	MMR vaccine	Immunization		2		
F	15	E, M, A	T, Su, St	Paracetamol	Fever	1	4	Yes	No
				Aciclovir	NR	NR	NR	No	NR
				Ceftibuten	NR	NR	NR	No	NR
F	18	E, M, A	T, Su	Ibuprofen	Rhinitis	1	1	Yes	No
				Beclomethasone	Rhinitis	4	2	Yes	No
M	28	E, M, A	T, Su	Calcarea 200 K	NR	79	2	Yes	No
				Omeos	Homeopathic vaccine	157	0	Yes	No
				Ribes drops	NR	4	0	Yes	No
F	33	M, A	T, Su, St	Paracetamol	Fever	3	0	Yes	Yes
				Ibuprofen	Fever	1	0	Yes	No
M	36	E, M, A	T, Su	Amoxicillin+ clavulanic acid	Pharyngotonsillitis	4	0	Yes	No
				Betamethasone	Tonsillitis	5	0	No	No
				Paracetamol	Tonsillitis	NR	NR	Yes	No
M	39	E, M, A	T, Su	Cefixime	URTI	6	1	No	No
				Beclomethasone	URTI	8	0	Yes	No
M	40	E, M, A	T, Su	Acetylcysteine	Common cold	7	1	Yes	No
				Beclomethasone	Common cold	7	1	Yes	No
				Salbutamol	Common cold	7	1	Yes	No
				Sobrerol	Cough/catarrh	8	1	Yes	No
				Cephaclor	Fever/Pharyngitis	5	8	Yes	No
F	45	E, M, A	T, Su, St	Clarithromycin	URTI	8	16	No	No
				Paracetamol	Fever	1	31	Yes	Yes
F	45	E, M	T, Su, St	Paracetamol+ chlorpheniramine	Tonsillitis	8	6	Yes	No
				Cetirizine	NR	4	3	Yes	No
				Levodropropizine	Tonsillitis	8	6	No	No
				Flu vaccine	Immunization		4		
F	48	E, M	T	Bacterial lysates	Prophylaxis URTI	10	0	Yes	No
				Epicutaneous patch testing (Hg)	Allergy diagnostic testing	4	0	No	No
M	53	E, M, A	T, Su	Azithromycin	URTI	21	6	No	No
				Desloratadine	URTI	21	7	No	No
				Mometasone	URTI	21	7	No	No
F	54	E, M	T, Su	No					
M	54	NA	NA	Phenobarbital	Prophylaxis fever convulsion	30	0	Yes	No
				Valproic acid	Prophylaxis fever convulsion	30	0	Yes	No
				Niflumic acid	Pharyngitis	3	7	Yes	No
				Paracetamol	Fever	11	0	Yes	No
M	83	E, M, A	T, Su	Clarithromycin	Fever-URTI	4	26	Yes	No
				Ceftriaxone	Fever-URTI	4	22	Yes	No
				Beclomethasone	Fever-URTI	3	NR	Yes	No
				Salbutamol	Fever-URTI	3	NR	Yes	No
				Budesonide	Fever-URTI	3	NR	Yes	No
				Valproic acid	Epilepsy	2044	0	Yes	Yes
				Clobazam	Epilepsy	1139	6	Yes	Yes
				Melatonin	Sedation	20	NR	Yes	No
M	89	M, A	T, Su, St	Phenobarbital	Convulsions	6	1	No	No
				Vitamins	Supplementation	5	3	No	No
F	108	E, M, A	T, Su, St	Ibuprofen	Fever	7	0	Yes	No
				Paracetamol	Fever	7	0	Yes	Yes
				Acetylcysteine	Cough	7	0	No	No
F	113	E, M, A	T, Su, St	Carbamazepine	Epilepsy	13	0	No	No
				Diazepam	Epilepsy	1	23	No	No
				Clotrimazole	Vaginal candidiasis	23	0	Yes	No
F	119	NA	NA	No					
F	123	M	T, Su	Lamotrigine	Epilepsy	25	0	No	No
				Paracetamol	Fever	9	0	Yes	No
				Valproic acid	Epilepsy	732	0	Yes	Yes
M	138	E, M	T, Su, St	Amoxicillin+ clavulanic acid	Fever-URTI	4	0	Yes	No
				Paracetamol	Fever	2	NR	Yes	No
				Beclomethasone	URTI	4	0	Yes	No
				Salbutamol	URTI	4	0	Yes	Yes
M	149	E, M, A	T, Su	Paracetamol	Pain	3	NR	Yes	Yes
				Amoxicillin+ clavulanic acid	Streptococcal infection	1	1	No	No

MMR vaccine: measles, mumps and rubella vaccine.

NR: not reported.

URTI: upper respiratory tract infection.

NA: clinical record not available.

The duration of drug use was generally short, with the exception of homeopathic remedies and food supplements. Nine children (out of the 23 with previous use of drugs) continued to receive, after admission, at least one of the drugs that were used prior to the hospitalization. All patients fully recovered from SJS or TEN.

Crude and adjusted ORs (adjustment by age and concomitant use of any other drugs) were estimated for all drugs with at least 2 exposed cases (Table 3). Antiepileptics presented the highest adjusted OR: 26.8 (95% CI 8.4–86.0). Within this category, the adjusted OR for valproic acid was 48.1 (95% CI 9.7–237.5), even though 1 out of the 3 exposed children recovered despite the treatment continued during hospitalization. The second largest risk was estimated for corticosteroids (adjusted OR: 4.2; 95% CI 1.8–9.9), followed by antibiotics (adjusted OR: 3.3; 95% CI 1.5–7.2), with overlapping confidence intervals of the various antibiotic categories. Significantly elevated risks were also estimated for paracetamol (adjusted OR: 3.2; 95% CI 1.5–6.9), but not for NSAIDs use (adjusted OR: 2.4; 95% CI 0.8–7.3).

**Table 3:** Odds ratio of SJS and TEN associated with drugs use.

Drugs	Cases	Controls*	Crude OR	Adjusted OR**
	(29)	(1,362)	(95% CI)	(95% CI)
Any Antibiotics	11	177	5.9 (2.1–16.1)	3.3 (1.5–7.2)
Any Penicillins	4	80	4.7 (1.3–17.1)	2.1 (0.7–6.3)
Amoxicillin+clavulanic acid	4	61	6.2 (1.7–22.5)	2.8 (0.9–8.4)
Any Cephalosporins	5	60	7.9 (2.3–26.5)	3.6 (1.3–9.9)
Cephaclor	2	16	11.8 (2.2–63.0)	4.9 (1.1–22.8)
Macrolides	3	36	7.9 (1.9–32.7)	3.2 (0.9–11.2)
Any Antiepileptics	5	13	36.3 (9.8–134.2)	26.8 (8.4–86.0)
Valproic acid	3	5	56.6 (11.0–292.3)	48.1 (9.7–237.5)
Any NSAIDs	4	88	4.3 (1.2–15.5)	2.4 (0.8–7.3)
Ibuprofen	3	44	6.4 (1.6–26.6)	3.3 (0.9–11.5)
Paracetamol	11	202	5.1 (1.9–14.1)	3.2 (1.5–6.9)
Paracetamol and antibiotics	6	56	10.1 (3.2–32.4)	5.1 (2.0–13.2)
Corticosteroids	9	108	7.9 (2.7–22.5)	4.2 (1.8–9.9)
Total drug use	23	796	2.7 (1.1–6.7)	2.4 (1.0–6.1)***
Non users	6	566		

* Neurological conditions.

** OR adjusted for age and concomitant use of other drugs.

*** OR adjusted for age.

Only minor changes were observed in the OR estimates when the sensitivity analysis was carried out by considering unexposed both cases and controls with drug use started in the index date or in the two previous days. Specifically, with regard to any drug use, 21 cases and 623 controls were still exposed (adjusted OR: 2.9; 95% CI 1.2–6.6). Negligible differences were also observed for drug use more sensitive to the shift in the exposure window: antibiotics (adjusted OR: 4.2; 95% CI 1.8–9.4); corticosteroids (adjusted OR: 4.1; 95% CI 1.7–10.1); paracetamol (adjusted OR: 2.9; 95% CI 1.3–6.5). No increased risk was observed for NSAIDs (adjusted OR: 1.2; 95% CI 0.3–5.5).

An increased risk was estimated for concomitant use of antibiotics and paracetamol (adjusted OR: 5.1; 95% CI 2.0–13.2). However, when the time window of exposure did not include the drugs assumed in the two days before the onset of symptoms the adjusted OR decreased to 3.9 (95% CI 1.3–11.9), similar to the risk estimates observed for antibiotics alone.

## Discussion

Our study provides additional evidence concerning the etiologic role of drugs and vaccines in the occurrence of SJS in children. In particular, an increased risk of twenty-seven times was estimated for antiepileptic drugs. A three times increased risk was observed for antibiotics, even though the limited power of the study does not allow to differentiate between the three main classes: penicillins, cephalosporins and macrolides. Among the other drugs, a statistically significant increased risk was observed for paracetamol and corticosteroids, with OR estimates ranging between 3.2 and 4.2.

Our findings are coherent with those of the pooled analysis of two international multicenter case-control studies [10] in which 80 children with SJS or TEN and 216 controls were analysed. All the estimates were generally lower in our study, even though entirely compatible when considering that the confidence intervals estimated in the international study were quite large, since either only one or no controls were exposed in many drug categories. For instance, the OR estimates associated with corticosteroids use were 5.6 (95% CI 0.8–∞) in the Levi’s study and 4.2 (95% CI 1.8–9.9) in ours.

The high proportion of cases exposed to at least one drug was similar to that reported by Levi et al. (79% vs 92%), despite a different definition of the risk period considered for the evaluation of exposure: 3 weeks preceding the index date in our study and 7 days in the Levi et al. An interval of 7 days after last exposure was considered consistent with the pharmacokinetics hypothesis that a drug could not induce any reaction if totally eliminated from the body [10]. In our study, with the exception of vaccines, the great majority of drug use was still occurring in the last week preceding the index day. The beginning of treatment is also of interest, and our time window for drug exposure is coherent with the natural history of SJS. For instance, the ALDEN (ALgorithm of Drug causality for Epidermal Necrosis) criteria for the causality assessment of drug-related SJS consider a delay from initial drug intake to the onset of the event as “suggestive” from 5 to 28 days, and “compatible” from 29 to 56 days [16].

Only one case of TEN was included in our study, whereas in the pooled case-control study 27 of the 80 cases (34%) received a diagnoses of TEN [10]. The discrepancy may be partly explained by the difference in the children’s age (a median of 3 years in our study and 6 years in the Levi et al.), since a better prognosis has been suggested for younger patients [17], [18].

We are aware of the difficulties in the diagnosis of SJS in the clinical practice, especially in children. However, the nine hospitals included in the Italian study are among the most renowned centers: five of them are paediatric hospitals (teaching and non-teaching hospitals) and the remaining four are teaching departments. The validation procedure was performed in almost all cases and only one of the two children whose clinical record was not retrieved was exposed to drugs. When the 8 cases with not confirmed diagnosis were included in a sensitivity analysis, no change was observed in the OR estimates.

A possible misclassification of more severe cases (TEN) should not bias the OR estimates, since SJS and TEN are considered as two variants of the same disease, whose occurrence is related to drug exposure in around 70% of the instances [9]. A reduction of the study power would be expected if more severe cases were lost in our surveillance. The possibility that some cases of erythema multiforme majus were classified as SJS and included as cases even after the validation process is unlikely. This misclassification would cause an underestimate of the ORs related to drug use, under the hypothesis that erythema multiforme were less strongly associated with drug exposure. However, erythema multiforme is one of the eligible conditions and the differential diagnosis with SJS is expected to be performed during the hospitalization. During the study period, 47 out of the 2,483 children who were hospitalized for muco-cutaneous conditions (1.8%) received a diagnosis of erythema multiforme.

It is unlikely that the diagnosis of SJS was influenced by the information on exposure since the drug anamnesis was ascertained by an independent monitor. It is also unlikely that the recall bias may explain the increased risks observed in our study since both cases and controls were hospitalized for acute conditions in the same setting. Furthermore, the study was performed without any predefined hypothesis and thus selection of cases associated with specific drug exposures can be reasonably excluded.

Despite the fact that only 29 children with SJS were enrolled, they represented the second largest, and probably the youngest (median age: 3 years) series of cases included in an observational study. Even though SJS is an extremely rare disease, given the severity of the condition it is important to further investigate possible associations with drugs. This will contribute to a more precise estimate on the role of drugs already implicated in the occurrence of SJS and confirm and/or highlight potential new signals.

In recent years, the discovery of the association between HLA alleles and some cutaneous ADRs (such as allopurinol-associated SJS and SJS associated with aromatic amine anticonvulsants) provided an important progress in knowledge [19]. Performing HLA and genome-wide analyses in children with severe cutaneous ADRs was not among the objectives of our study. However, current surveillance systems, together with new observational studies in children, may be encouraged to verify the hypothesis of genetic variation leading to altered metabolism of drugs, especially for category of drugs as anticonvulsants that are targeted at well defined groups of patients. The general rule of assessing the benefit/risk of each treatment and avoiding inappropriate prescriptions will remain the safest behavior to reduce the occurrence of SJS related to drugs of more common use.

## Acknowledgments

**Italian multicenter study group for drug and vaccine safety in children.**

Francesca Menniti- Ippolito, Roberto Da Cas, Luciano Sagliocca, Giuseppe Traversa (National Center for Epidemiology, National Institute of Health, Roma, Italy); Fernanda Ferrazin, Carmela Santuccio, Loriana Tartaglia, Francesco Trotta (Italian Medicines Agency, Roma, Italy); Pasquale Di Pietro, Salvatore Renna, Rossella Rossi, Bianca Domenichini, Stefania Gamba, Francesco Trovato (Giannina Gaslini Paediatric Hospital, Genova, Italy); Pier-Angelo Tovo, Manuela Bianciotto, Carmelina Calitri, Clara Gabiano, Irene Raffaldi, Antonio Urbino (Regina Margherita Paediatric Hospital, Torino, Italy); Liviana Da Dalt, Valentina Favero, Laura Giordano, Maura Baraldi, Federica Bertuola, Eleonora Lorenzon, Francesca Parata, Giorgio Perilongo, Silvia Vendramin (Department of Paediatrics, University of Padova, Italy); Monica Frassineti, Anna Maria Calvani, Elena Chiappini, Maurizio De Martino, Claudia Fancelli, Francesco Mannelli, Rachele Mazzantini, Sara Sollai, Elisabetta Venturini (Anna Meyer Children’s University Hospital, Firenze, Italy); Mario Furbetta, Piera Abate, Ilaria Leonardi (Department of Paediatrics, University of Perugia, Italy); Nicola Pirozzi, Umberto Raucci, Antonino Reale, Rossella Rossi (Paediatric Emergency Department, Bambino Gesù Children Hospital, Roma, Italy); Nadia Mores, Giulia Bersani, Adele Compagnone, Antonio Chiaretti, Riccardo Riccardi, Costantino Romagnoli (Pharmacology and Paediatrics, Università Cattolica S. Cuore, Roma, Italy); Vincenzo Tipo, Michele Dinardo, Fabiana Auricchio (Santobono Paediatric Hospital, Napoli, Italy); Annalisa Capuano, Alessandra Maccariello, Elisabetta Parretta, Concita Rafaniello (Department of Experimental Medicine, Section of Pharmacology “L. Donatelli”, Second University of Napoli, Italy); Fortunata Fucà, Eleonora Di Rosa (Giovanni Di Cristina Paediatric Hospital, Palermo, Italy).
